# Point-of-Care Ultrasound for Suspected Radiographically Occult Proximal Femoral Fracture in Adults With Acute Hip Pain: A Systematic Review of Diagnostic Accuracy Studies

**DOI:** 10.7759/cureus.117910

**Published:** 2026-10-14

**Authors:** Omer Kamal Ahmed Omer, Haitham Abdallah, Ahmed Kamaleldin Abdelmahmoud Salman, Elsheimaa Mohamedsalih Osman Mohamedsalih, Amgad Abdelhadi Elhag Abdalla, Aaraf Alsayed Albasheer Adam, Rasha Omer Babiker Mohamed, Wafi Mohammed, Alaa Albadwy

**Affiliations:** 1 Trauma and Orthopaedics, Luton and Dunstable Hospital, Luton, GBR; 2 Emergency Medicine, Global Care Hospital, Abu Dhabi, ARE; 3 Accident and Emergency, Emirates Health Services-Khorfakkan Hospital, Sharjah, ARE; 4 Accident and Emergency, University Hospital Limerick, Limerick, IRL; 5 Accident and Emergency, Barnsley Hospital NHS Foundation Trust, Barnsley, GBR; 6 Emergency Medicine, Gloucestershire Royal Hospital, Gloucestershire, GBR; 7 Trauma and Orthopaedics, Nobles Hospital, Douglas, IMN; 8 Trauma and Orthopaedics, South Tees Hospitals NHS Foundation Trust, Middlesbrough, GBR; 9 Internal Medicine, Almanagil Teaching Hospital, Almanagil, SDN

**Keywords:** diagnostic accuracy, emergency department, femoral neck fracture, musculoskeletal ultrasound, occult hip fracture, point-of-care ultrasound, systematic review

## Abstract

Radiographically occult proximal femoral fracture is a recognised diagnostic hazard in acute care. Magnetic resonance imaging (MRI) is the reference standard but is rarely available at the bedside, and point-of-care ultrasound (POCUS) has been proposed as an adjunct that may modify clinical suspicion while advanced imaging is arranged. This systematic review aimed to identify and appraise primary studies of clinician-performed hip ultrasound in adults presenting acutely with hip pain and suspected proximal femoral fracture with non-diagnostic or pending radiographs.

PubMed/MEDLINE, Embase, Scopus, Web of Science, the Cochrane Library and trial registers were searched from inception to 1 September 2026, with citation searching. The review was reported according to the Preferred Reporting Items for Systematic Reviews and Meta-Analyses (PRISMA) 2020 and PRISMA-diagnostic test accuracy (PRISMA-DTA) and was not prospectively registered. Eligible studies were original peer-reviewed diagnostic accuracy studies using B-mode hip ultrasound against MRI, computed tomography (CT) or a composite reference standard; radiography was accepted only to confirm a visible fracture, never to classify a participant as fracture-negative. Two-by-two tables were reconstructed, exact binomial confidence intervals (CIs) were derived, and risk of bias was appraised with the Quality Assessment of Diagnostic Accuracy Studies, Version 2 (QUADAS-2). Pooling was not performed because of clinical and methodological heterogeneity.

Four prospective studies (389 participants; 186 with proximal femoral fracture) were included. Only two restricted enrolment to radiograph-negative patients and verified all participants with MRI. Observed sensitivity ranged from 0.96 to 1.00, with lower CI bounds of 0.69-0.92. Specificity ranged from 0.65 to 0.99; the lowest values occurred with qualitative capsular signs and the highest with cortical discontinuity or a measured capsular threshold, but these were indirect comparisons confounded by setting, operator, population and reference standard. A hypothetical scenario analysis suggested that the largest study's sensitivity would fall to 0.89 if only 10 fractures had been missed among unverified excluded patients. Every study was at high or unclear risk of bias in at least one QUADAS-2 domain, and fracture-subtype-specific accuracy could not be reconstructed.

Hip POCUS showed high observed sensitivity and variable specificity in small, heterogeneous and biased studies; these estimates should not be taken as its true performance in routine emergency practice. The evidence supports POCUS only as an adjunct that may modify suspicion and help prioritise CT or MRI, not as a rule-out test. Adequately powered emergency department studies with blinded emergency-clinician sonographers, prespecified criteria, subtype-specific reporting and universal MRI verification are needed.

## Introduction and background

Fragility fracture of the proximal femur is among the most consequential injuries encountered in emergency medicine, and plain radiography remains the first-line investigation. Radiography is nonetheless imperfect. In emergency department (ED) patients with hip pain and negative initial radiographs, a clinically important minority were later shown to have a hip or pelvic fracture [1]. A pooled analysis of elderly patients with persisting suspicion despite negative radiographs found a surgically relevant fracture in a substantial proportion [2]. Operative delay in older patients has long been associated with more complications and higher mortality [3], and an unrecognised undisplaced fracture may displace on mobilisation [4].

Magnetic resonance imaging (MRI) is the reference standard for radiographically occult fracture, detecting marrow oedema and fracture lines invisible on radiographs [4,5]. Computed tomography (CT) is more accessible out of hours but misses a proportion of undisplaced and trabecular-only injuries [5-7]. Current guidance therefore recommends MRI when suspicion persists after negative radiographs, with CT reserved for when MRI is unavailable or contraindicated [8,9]. Both modalities require transfer of a frail patient and competition for scanner time, so decisions about analgesia, weight-bearing and referral are often made hours before definitive imaging.

The target condition needs anatomical precision. Femoral head and femoral neck fractures are intracapsular, whereas intertrochanteric and most trochanteric-region fractures are extracapsular. An intracapsular fracture is expected to produce haemarthrosis that distends the anterior capsule; an extracapsular fracture may not. The diagnostic meaning of capsular distension therefore cannot be assumed to be the same across all proximal femoral fracture types. Femoral head fractures are rare after low-energy falls and are included in the target condition for completeness, but no subtype-specific evidence for them was expected.

Point-of-care ultrasound (POCUS) is already embedded in emergency care for trauma assessment, procedural guidance and regional anaesthesia and has established accuracy for fractures at several appendicular sites, with pooled sensitivity and specificity for lower-limb fractures in adults of approximately 0.83 and 0.93 [10]. The proximal femur is a more demanding target: it lies deep, only the anterior cortex is reliably insonated, and the probe and settings must suit variable body habitus. Two categories of signs have been proposed. A direct sign is a step, gap or irregularity of the femoral cortex. Indirect signs are soft-tissue consequences of injury: anterior capsular distension, joint effusion or iliopsoas haematoma. The visibility of each is likely to vary with fracture location, accessible cortical surface, body habitus, probe and operator. A published case illustrates the potential of the technique [11]. Its intended role is as an adjunct that may modify suspicion while CT or MRI is arranged, not as a replacement for advanced imaging.

The primary literature has not been formally synthesised, and few studies are restricted to confirmed radiograph-negative patients. This review therefore addressed the broader population of adults with acute hip pain and suspected proximal femoral fracture in whom ultrasound was applied before or at the point that radiographic occultness was established while reporting separately which studies addressed the occult-fracture question directly. The objectives were to identify eligible diagnostic accuracy studies of clinician-performed hip ultrasound, describe the sonographic criteria and techniques evaluated, summarise diagnostic performance and appraise risk of bias and applicability to emergency practice.

## Review

Materials and methods

Protocol and Reporting

This review was conducted and reported according to the Preferred Reporting Items for Systematic Reviews and Meta-Analyses (PRISMA) 2020 [12] and its extension for diagnostic test accuracy reviews, PRISMA-DTA [13]. The review was not prospectively registered in the International Prospective Register of Systematic Reviews (PROSPERO) or elsewhere; this is a major limitation, discussed below. Ethical approval was not required because only published aggregate data were used.

Eligibility Criteria

Eligibility was defined a priori using the PICOS framework described by Liberati et al. [14] (Table 1). Because few studies were expected to be restricted to confirmed non-diagnostic radiographs, the population included adults under acute suspicion of proximal femoral fracture in whom ultrasound was applied at or before the point at which radiographic occultness was established. Studies were classified a priori as either occult-restricted (enrolment limited to radiograph-negative patients) or suspected-fracture cohorts, and this classification is carried through every result.

**Table 1:** Eligibility criteria structured according to the PICOS framework ED: emergency department; MRI: magnetic resonance imaging; CT: computed tomography

PICOS element	Inclusion criteria	Exclusion criteria
Population	Adults (18 years or older) presenting acutely with hip pain, usually after low-energy trauma, in an ED or equivalent unscheduled acute-care setting; clinical suspicion of proximal femoral fracture (femoral head, neck, intertrochanteric or trochanteric); non-diagnostic radiographs or ultrasound applied at or before the point radiographic occultness was established	Exclusively paediatric cohorts; asymptomatic volunteers; elective, preoperative or postoperative populations; high-energy polytrauma where hip pain was not the presenting complaint; ipsilateral arthroplasty or previous ipsilateral hip surgery
Index test	B-mode ultrasound of the symptomatic hip by a clinician or radiologist, any device class, targeting cortical discontinuity (direct sign) and/or capsular distension, effusion or iliopsoas haematoma (indirect signs)	Ultrasound used only to guide regional anaesthesia or aspiration; quantitative bone ultrasound; Doppler-only, contrast or elastography protocols; other skeletal sites
Reference standard	MRI, CT or a composite of imaging and documented clinical or surgical follow-up; radiography accepted only to confirm a radiographically visible fracture	Radiography alone used to classify participants as fracture-negative; reference standard chosen on the basis of the ultrasound result; reference standard not reported
Outcomes	Primary: sensitivity and specificity for proximal femoral fracture. Secondary: predictive values, likelihood ratios, reconstructable two-by-two counts, accuracy by fracture subtype, inter-observer reliability, examination time, downstream management outcomes	Descriptive, technical or feasibility reports without accuracy data or a reconstructable two-by-two table
Study design	Original peer-reviewed prospective or retrospective diagnostic accuracy, diagnostic cohort or cross-sectional studies	Reviews and meta-analyses; case reports and non-comparative case series; conference abstracts, editorials, letters, protocols, registry entries and pre-prints; animal, cadaveric and phantom studies
Other	Full text available; English language; peer-reviewed journal; no restriction on year or country	Full text unobtainable; non-English; duplicate reports of one cohort (most complete retained)

The handling of plain radiography was specified to prevent the misclassification of the target condition. Radiography was accepted only to confirm a fracture that was visible on it. A participant could be classified as fracture-negative only on the basis of MRI, CT or a composite of imaging with documented clinical follow-up. Studies in which radiograph-negative participants were classified as fracture-free on radiography alone were ineligible.

Information Sources and Search Strategy

PubMed/MEDLINE, Embase, Scopus, Web of Science Core Collection and the Cochrane Central Register of Controlled Trials were searched from inception to 1 September 2026. ClinicalTrials.gov and the WHO International Clinical Trials Registry Platform were searched for completed but unpublished work. The search combined controlled vocabulary and free text across three concept blocks: proximal femoral fracture, ultrasound and diagnostic accuracy. No date, design, language or geographical limits were applied at the search stage. The complete database-specific strategies, including platform, search date and records retrieved, are given in the Appendices. Reference lists of included studies and relevant reviews were hand-searched, and forward citation tracking was performed in Google Scholar and Scopus. Because fewer than 10 studies were eligible, the prespecified contingency of restricting inclusion to the preceding five years was not applied.

Study Selection

Records were de-duplicated electronically and then manually. Title and abstract screening and full-text assessment were performed by one reviewer (HA) in two passes separated by two weeks, with discrepancies resolved by returning to the full text. A second reviewer (ROBM) independently re-assessed all 45 full-text reports and verified the final inclusion decisions; disagreements were resolved by discussion. Non-English and grey-literature records were excluded at the stage they were identified; their number is reported in the Results.

Data Extraction

Data were extracted by a reviewer (HA) into a piloted standardised form and independently verified cell by cell by a second reviewer (ROBM). Extracted items were as follows: study identifiers, design, setting and recruitment; eligibility criteria; sample size, age and sex; whether enrolment was occult-restricted; fracture subtype distribution (femoral head, neck, intertrochanteric/trochanteric, other); device, transducer type (linear or curvilinear), frequency, footprint, depth and focal settings, patient position, scanning planes and anatomical targets, use of different probes for cortical and capsular assessment, contralateral comparison and frequency of inadequate visualisation; the operational definition of each positive criterion and whether cortical findings were confirmed in two orthogonal planes; operator specialty, experience, training and blinding; the reference standard applied to each participant group and its timing; and all reported accuracy measures. Items not reported are stated as not reported.

Reconstruction of Two-by-Two Tables

Cell counts were taken directly from the published text or tables where available. Otherwise, true positives were calculated as sensitivity multiplied by the number of reference-positive participants and true negatives as specificity multiplied by the number of reference-negative participants, each rounded to the nearest integer; false negatives and false positives were obtained by subtraction. Each reconstructed table was checked against every other published estimate (predictive values, likelihood ratios, prevalence). Exact Clopper-Pearson 95% confidence intervals (CIs) were calculated from the beta distribution. Where a recalculated estimate differed from the published value, both are reported and the reason is given.

Risk of Bias and Applicability

Risk of bias and applicability were appraised with the Quality Assessment of Diagnostic Accuracy Studies, Version 2 (QUADAS-2) [15] by a reviewer (HA) and verified by a second reviewer (ROBM). Signalling questions were answered from the published text, and a domain was rated some concerns only when information was insufficient for a judgement.

Data Synthesis

Meta-analysis was considered and rejected. The bivariate random-effects and hierarchical summary receiver operating characteristic (HSROC) models require several studies evaluating a common index test definition; here, each sonographic criterion was evaluated in only one or two studies, positivity thresholds differed, and two studies were at high risk of bias in the reference standard or flow and timing domains. Findings are therefore synthesised narratively, grouped first by study population (occult-restricted versus suspected-fracture) and then by sonographic criterion, with individual estimates and exact CIs. Accuracy by fracture subtype was prespecified but is reported only where data were extractable. To explore partial verification, a scenario analysis recalculated sensitivity in the largest study under assumed numbers of missed fractures among unverified, excluded participants.

Results

Study Selection

Database searches returned 1202 records. After removing 442 duplicates, 760 records were screened and 713 excluded. Of 47 reports sought, 45 were retrieved and assessed in full; two were unobtainable. Forty-one were excluded: 12 reviews, nine case reports or series, eight with an ineligible index test (mostly ultrasound-guided nerve blockade or other skeletal sites), four paediatric, five conference abstracts, protocols or registry entries and three without extractable accuracy data. Citation searching identified 14 further reports, of which six were assessed in full and all excluded because two of them were based on a paediatric population and four were abstracts, protocols and registry entries. Four studies were included (Figure 1).

**Figure 1:**
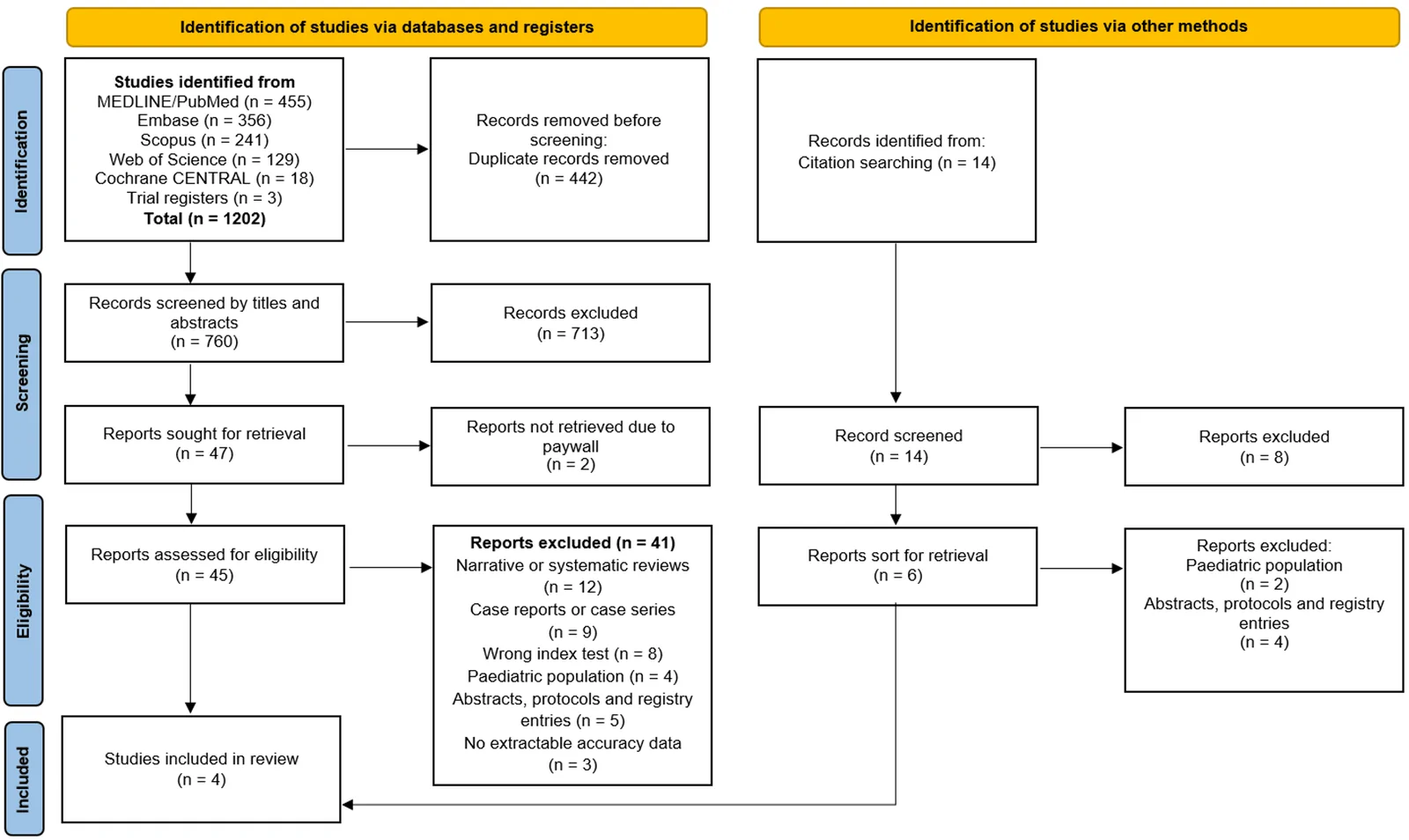
PRISMA 2020 flow diagram showing identification via databases and registers and via other methods (citation searching) PRISMA: Preferred Reporting Items for Systematic Reviews and Meta-Analyses

Characteristics of the Included Studies

The four prospective studies were published between 2009 and 2023 and enrolled 389 participants, 186 (47.8%) with a proximal femoral fracture [16-19]. Two restricted enrolment to radiograph-negative patients and verified every participant with MRI and therefore address the occult-fracture question directly [16,18]. The other two enrolled patients under general suspicion, so their estimates include radiographically visible fractures [17,19]; in the larger, 13 of 213 radiographic readings were later shown to be falsely negative [19]. Only one study [19] was conducted wholly in EDs. Cohorts were elderly and predominantly female (Table 2).

**Table 2:** Characteristics of the included studies ED: emergency department; MRI: magnetic resonance imaging; CT: computed tomography; NR: not reported

Study	Country	Setting	Population	Occult-restricted	n	Fractures, n (%)	Fracture subtype	Reference standard
Safran et al., 2009 [16]	Israel	Inpatients admitted after emergency presentation	Painful hip after low-energy trauma, non-diagnostic radiographs; mean age 73 years	Yes	30	10 (33.3)	NR	MRI in all participants
Akimoto et al., 2020 [17]	Japan	Emergency outpatient unit (primary care)	Acute hip pain; mean age 78.0 years; 88.5% female	No	52	26 (50.0)	NR	Variable between participants (imaging and follow-up)
Tsukamoto et al., 2023 [18]	Japan	University orthopaedic outpatient service	Age over 70 years, acute hip pain, no radiographic abnormality; mean age 81.8 years; 88.3% female	Yes	94	27 (28.7)	Femoral neck only	MRI in all participants
Cohen et al., 2023 [19]	United States	Two academic EDs	High clinical suspicion of hip fracture; convenience sample	No	213	123 (57.7)	NR	Radiography for radiographically visible fractures; CT or MRI for all radiograph-negative participants (those without it excluded)

Sonographic Technique and Criteria

Technical reporting was poor (Table 3). Only one study reported the transducer, a linear probe on a pocket-sized device [17]; transducer type was not reported in the other three. Frequency, footprint, depth and focal settings, the frequency of inadequate visualisation and the influence of body habitus were not reported in any study. No study prespecified how an acute cortical interruption was distinguished from old fracture, degenerative irregularity, osteophyte, enthesopathy, an anatomical interface or artefact, and none reported confirmation in two orthogonal planes. This is a major reproducibility and applicability gap: linear and curvilinear probes are not interchangeable for the deep anterior femoral neck in elderly patients.

**Table 3:** Sonographic technique, criteria and operators NR: not reported; ROC: receiver operating characteristic; ED: emergency department

Study	Device; transducer	Scanning approach	Positive criterion	Contralateral comparison	Operator; blinding
Safran et al., 2009 [16]	Cart-based; transducer NR	Symptomatic and contralateral hips	Qualitative trauma-related change, including capsular distension and effusion (indirect)	Yes	Two musculoskeletal radiologists
Akimoto et al., 2020 [17]	Vscan Dual Probe pocket device (GE HealthCare, Chicago, IL, USA); linear	Four-step sequence: femoral shaft short axis, proximal slide, rotation at lesser trochanter, femoral neck long axis	Cortical discontinuity (direct); joint fluid as capsule-to-neck distance over 6 mm (indirect)	NR	One physician, 11 years' ultrasound experience; not blinded to clinical findings
Tsukamoto et al., 2023 [18]	Conventional B-mode; transducer NR	Long axis of the femoral neck	Capsule-to-neck distance of 5.3 mm or more, derived by ROC curve analysis in the same sample (indirect)	NR	Orthopaedic assessors
Cohen et al., 2023 [19]	ED systems; transducer NR	Anterior hip and iliopsoas region	Qualitative hip effusion and/or iliopsoas haematoma (indirect)	NR	19 sonographers: 10 emergency ultrasound fellows, 9 fellowship-trained physicians; blinded to ED imaging

Diagnostic Performance

Reconstructed two-by-two data are shown in Table 4 and accuracy estimates in Table 5 and Figure 2.

**Table 4:** Two-by-two data Tsukamoto et al. [18] reported specificity as 0.98; the reconstructed 66/67 is 0.985, consistent with truncation rather than rounding. TP: true positive; FP: false positive; FN: false negative; TN: true negative; LR: likelihood ratio

Study	Criterion	Target condition	n	TP	FP	FN	TN	Derivation
Safran et al., 2009 [16]	Qualitative capsular/trauma-related change	Hip fracture	30	10	7	0	13	Counts reported in text
Tsukamoto et al., 2023 [18]	Capsule-to-neck distance of 5.3 mm or more	Femoral neck fracture	94	26	1	1	66	Derived from reported sensitivity and specificity and group sizes
Akimoto et al., 2020 [17]	Cortical discontinuity	Proximal femoral fracture	52	25	2	1	24	TP and FP reported; FN and TN by subtraction
Akimoto et al., 2020 [17]	Cortical discontinuity or joint fluid	Composite: fracture or acute hip arthritis	52	31	2	1	18	As above; not a fracture-only estimate
Cohen et al., 2023 [19]	Qualitative effusion and/or iliopsoas haematoma	Hip fracture	213	119	27	4	63	Derived; reproduces published LR+ 3.22 and LR- 0.05

**Table 5:** Diagnostic performance LRs were calculated by the review except those of Cohen et al. [19], which match the published values (published 95% CIs: LR+ 2.35-4.43; LR- 0.02-0.12). The LR+ of Tsukamoto et al. [18] rests on a single false positive and is highly unstable. ^†^Safran et al. [16] had no false negatives, so the observed LR− is 0; its CI cannot be estimated without a continuity correction (with 0.5 added to each cell, LR− = 0.07). This observed zero reflects a small sample with 10 fractures and does not demonstrate reliable rule-out performance. CI: confidence interval; PPV: positive predictive value; NPV: negative predictive value; LR: likelihood ratio

Study	Sensitivity (95% CI)	Specificity (95% CI)	PPV (95% CI)	NPV (95% CI)	LR+	LR-
Occult-restricted, universal MRI						
Safran et al., 2009 [16]	1.00 (0.69-1.00)	0.65 (0.41-0.85)	0.59 (0.33-0.82)	1.00 (0.75-1.00)	2.86	0 (observed)^†^
Tsukamoto et al., 2023 [18]	0.96 (0.81-1.00)	0.99 (0.92-1.00)	0.96 (0.81-1.00)	0.99 (0.92-1.00)	64.5	0.04
Suspected-fracture cohorts						
Akimoto et al., 2020 [17], cortical discontinuity	0.96 (0.80-1.00)	0.92 (0.75-0.99)	0.93 (0.76-0.99)	0.96 (0.80-1.00)	12.5	0.04
Akimoto et al., 2020 [17], composite target	0.97 (0.84-1.00)	0.90 (0.68-0.99)	0.94 (0.80-0.99)	0.95 (0.74-1.00)	9.69	0.03
Cohen et al., 2023 [19]	0.97 (0.92-0.99)	0.70 (0.59-0.79)	0.82 (0.74-0.87)	0.94 (0.85-0.98)	3.22	0.05

**Figure 2:**
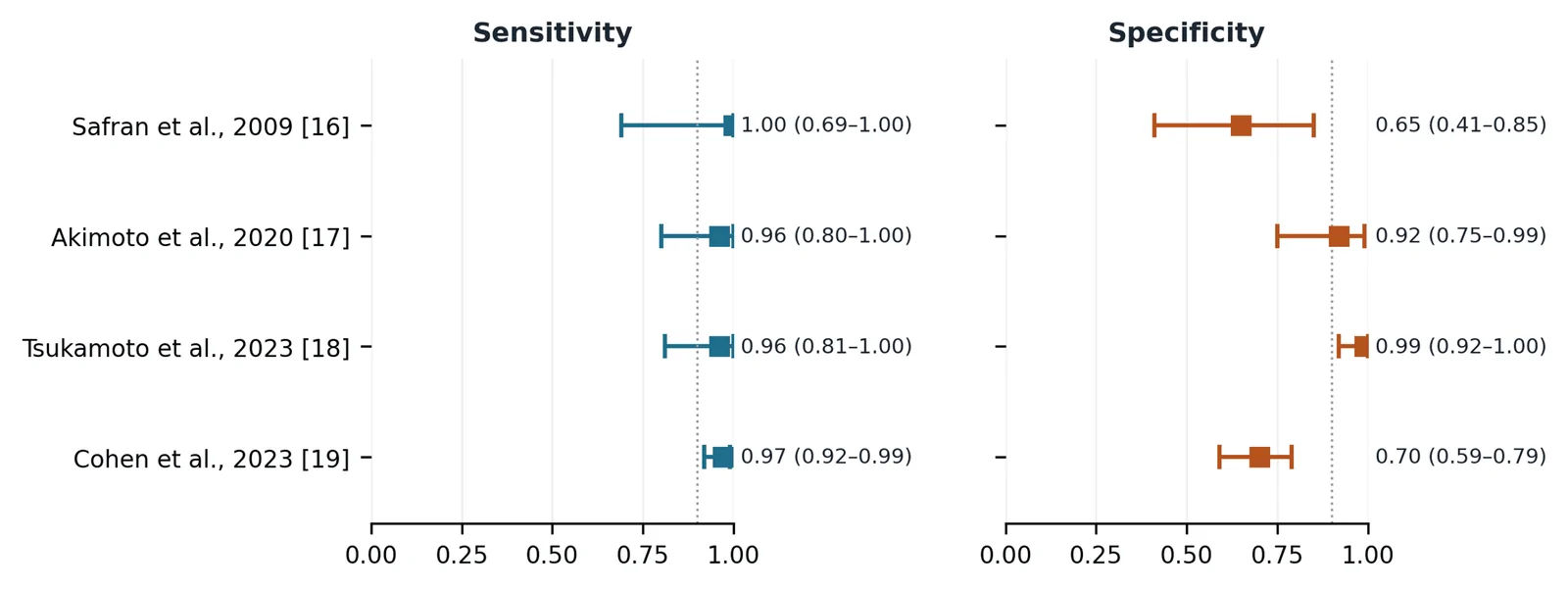
Sensitivity and specificity of hip ultrasonography for proximal femoral fracture (fracture-only estimates) Squares denote point estimates and horizontal lines exact (Clopper-Pearson) 95% confidence intervals.

Observed sensitivity ranged from 0.96 to 1.00, but CIs were wide in the three smaller studies, with lower bounds of 0.69-0.81. In the two occult-restricted studies with universal MRI, sensitivity was 1.00 (0.69-1.00) and 0.96 (0.81-1.00). These are the least biased estimates available, but together they rest on 37 fractures. In the two suspected-fracture cohorts, estimates include radiographically visible, often displaced fractures that are expected to be easier to detect sonographically, so they likely overstate sensitivity for occult fracture.

Specificity ranged from 0.65 to 0.99. The two lowest values came from qualitative indirect signs (0.65 [16]; 0.70 [19]) and the two highest from cortical discontinuity (0.92 [17]) and a measured capsular threshold (0.99 [18]). This pattern is an indirect, between-study comparison: the criterion was confounded with setting, operator, population, fracture subtype and reference standard, so it cannot be attributed to the criterion alone. In Safran et al., three of seven false positives had pubic fractures [16]. In Tsukamoto et al., the mean capsule-to-neck distance was 7.53 ± 1.52 mm with occult femoral neck fracture and 3.45 ± 0.89 mm without [18]; the threshold was derived and tested in the same sample, so its specificity is likely optimistic through overfitting.

Akimoto et al. evaluated the combined criterion of cortical discontinuity or joint fluid against a composite target of fracture or acute hip arthritis, with 32 target-positive participants rather than 26 fractures [17]. This estimate cannot show that direct and indirect signs are complementary for fracture diagnosis and is not used for fracture inference.

Accuracy by Fracture Subtype

Subtype-specific accuracy could be extracted only from Tsukamoto et al., whose cohort was restricted to femoral neck fractures [18]. No study reported accuracy separately for intertrochanteric or trochanteric fractures or for femoral head fractures, and the required two-by-two data were unavailable. Pooled observations therefore cannot show whether capsular signs perform equally in intracapsular and extracapsular fractures.

Scenario Analysis for Partial Verification

Cohen et al. excluded radiograph-negative patients who did not undergo CT or MRI [19]. If any excluded patient had an undetected fracture with a negative ultrasound, observed sensitivity is inflated. Assuming 5, 10 or 20 such missed fractures, sensitivity would fall from 0.97 to 0.93 (0.87-0.97), 0.89 (0.83-0.94) and 0.83 (0.76-0.89), respectively. This analysis is hypothetical: it illustrates how sensitive the estimate is to unverified cases and is not an empirical correction of the original data. A small number of unverified missed fractures would thus move sensitivity below the range commonly regarded as adequate for rule-out.

Risk of Bias and Applicability

No study was at low risk of bias in all four QUADAS-2 domains (Figure 3). Patient selection was at high risk in the convenience sample [19] and some concerns in two studies that did not state consecutive enrolment [16,17]. The index test domain was at high risk where a single examiner was not blinded to the clinical presentation [17] and where the threshold was derived within the evaluation sample [18]. The reference standard was at high risk in Cohen et al. because radiography confirmed most fracture-positive participants, so the condition verified was largely radiographically visible fracture rather than the occult target [19]. Flow and timing was at high risk in that study because of partial verification [19] and some concerns where the reference standard varied between participants [17].

**Figure 3:**
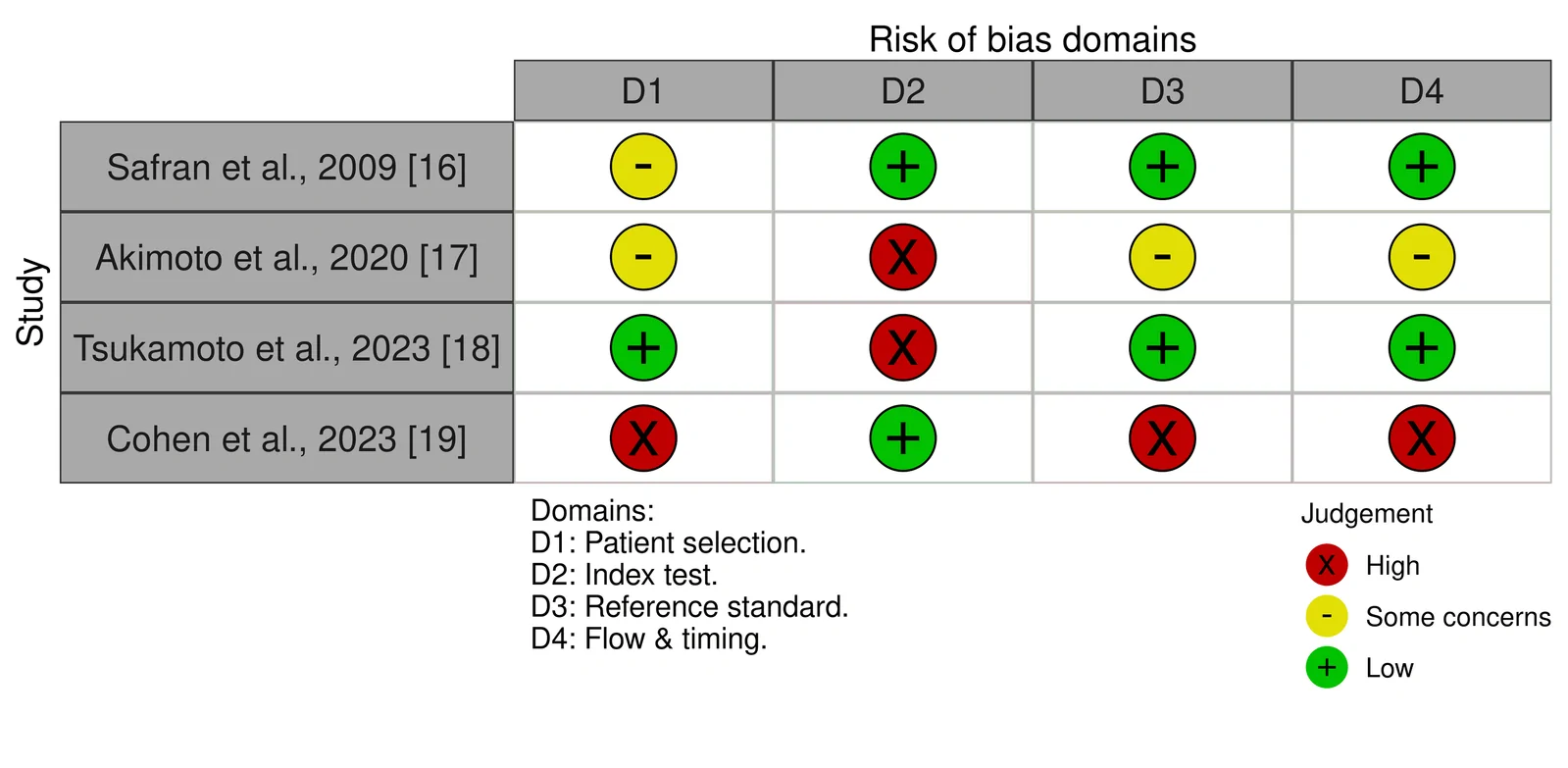
QUADAS-2 summary of risk of bias and concerns regarding applicability for each included study QUADAS-2: Quality Assessment of Diagnostic Accuracy Studies, Version 2

Applicability concerns were at least as important. Patient selection raised high concern in three studies, because the setting was not an ED [18] or the population was not occult-restricted [17,19]. The index test raised high concern where scans were done by musculoskeletal radiologists [16] or orthopaedic specialists [18]. Only Cohen et al. used operators typical of emergency POCUS, and that study carried the greatest concern in the reference standard and flow domains [19] (Table 6).

**Table 6:** Summary of findings ED: emergency department; MRI: magnetic resonance imaging; CT: computed tomography

Finding	Evidence	Certainty	Implication
Observed sensitivity is high	0.96-1.00 in four studies; occult-restricted, MRI-verified studies 1.00 and 0.96 (37 fractures)	Low: small samples, wide CIs, spectrum and verification bias	A normal scan does not exclude fracture
Specificity differs between studies	0.65 and 0.70 with qualitative indirect signs; 0.92 with cortical discontinuity; 0.99 with a measured threshold	Very low: single-study estimates, indirect comparison, confounded	Prespecified, measured criteria should be tested prospectively
Capsular distension is measurable	7.53 versus 3.45 mm [18]; a 6 mm cut-off used elsewhere [17]	Low: threshold derived in-sample	A 5-6 mm threshold needs external validation
Non-hip pelvic injury causes false positives	Three of seven false positives were pubic fractures [16]	Low	A positive capsular sign without a cortical break warrants pelvic imaging
Subtype-specific accuracy is unknown	Only femoral neck data [18]	No evidence for extracapsular or femoral head fractures	Capsular signs may underperform in extracapsular fractures
Ultrasound cannot replace advanced imaging	Two studies verified all participants with MRI, but both were small and used radiologists or orthopaedic assessors outside EDs [16,18]; no study combined an ED cohort, emergency-clinician operators and universal MRI	Consistent gap	CT or MRI remains indicated when suspicion persists, whatever the ultrasound result
Downstream impact is unstudied	No data on time to imaging or surgery, length of stay, opioid use or missed fractures	No evidence	Impact studies are needed before pathway adoption

Discussion

Principal Findings

This review identified four small diagnostic accuracy studies (389 adults) of hip ultrasound for suspected proximal femoral fracture. Observed sensitivity was 0.96-1.00 and specificity 0.65-0.99. These are observed values from selected, high-prevalence cohorts with spectrum, selection and verification bias; they should not be read as the true sensitivity of POCUS in routine ED patients with radiographically occult fracture. The two studies that addressed the occult question directly and verified everyone with MRI were small (37 fractures in total) and used radiologists or orthopaedic assessors outside the ED [16,18].

Direct and Indirect Signs and Fracture Location

Two different targets have been grouped under hip POCUS. Cortical discontinuity depicts the fracture itself, whereas capsular distension, effusion and iliopsoas haematoma depict its consequences. A cortical sign may be more specific than a capsular sign, but this rests on one small study [17] in which both false-positive and false-negative findings occurred. Cortical irregularity in older adults is also common for other reasons, and future protocols should classify it as acute fracture-related, old fracture, degenerative or osteophytic, enthesopathic, a normal anatomical interface or artefact, with confirmation in two orthogonal planes and correlation with CT or MRI. Without such a classification, pre-existing abnormalities may be misread as acute fractures.

Indirect signs are non-specific: effusion also arises from osteoarthritis, synovitis and pubic, ischial or trochanteric injury, all common in older patients after a fall [16]. Their meaning should also differ by fracture location. Intracapsular femoral neck fractures are expected to cause haemarthrosis, whereas extracapsular intertrochanteric fractures may not distend the capsule. The only subtype-specific data come from femoral neck fractures [18], so the performance of capsular criteria in extracapsular fractures is unknown. The between-study differences in specificity may reflect the criterion, but they equally reflect differences in operator expertise, probe, setting, population, fracture mix and reference standard.

Apparent sensitivity in the suspected-fracture cohorts is best explained by spectrum and verification effects, not by prevalence itself. Including radiographically visible and displaced fractures enriches the sample with easier cases, and excluding unverified radiograph-negative patients removes the cases most likely to be missed [17,19]. Both inflate observed sensitivity.

Comparison With Existing Literature

POCUS performance generally falls as anatomical depth increases. Weinberg et al. reported a sensitivity of 73% and specificity of 92% for clinician-performed fracture POCUS in children and young adults, with most errors at bone ends [20]. Superficial sites such as the ankle and foot perform best [10,21]. Against this background, observed hip sensitivity near 0.96 is unexpectedly high, which supports caution about bias. Capsular distension on ultrasound correlates with intra-articular effusion and synovitis [22], and a radiographic analogue, fat pad elevation of 1.5 mm or more, identified occult femoral neck fracture with 0.87 sensitivity and 0.86 specificity [23]. Both suggest that measured rather than qualitative capsular criteria deserve testing. Dual-energy CT [24] and artificial intelligence applied to radiographs [25] are emerging alternatives, but both require the patient to leave the bedside.

Clinical Implications and Proposed Pathway

The data support only an adjunctive role (Figure 4). After negative or equivocal radiographs with persisting suspicion, POCUS may be performed while CT or MRI is arranged, in line with current guidance [8,9]. A positive scan should expedite advanced imaging, non-weight-bearing precautions and early orthopaedic consultation. A negative scan must not end the work-up: CT or MRI should proceed when persistent pain, inability to bear weight or other findings maintain suspicion. Ultrasound is not a mandatory gatekeeper. A negative likelihood ratio of 0.05 [19] was generated mostly against radiographically visible fractures with partial verification and cannot support rule-out.

**Figure 4:**
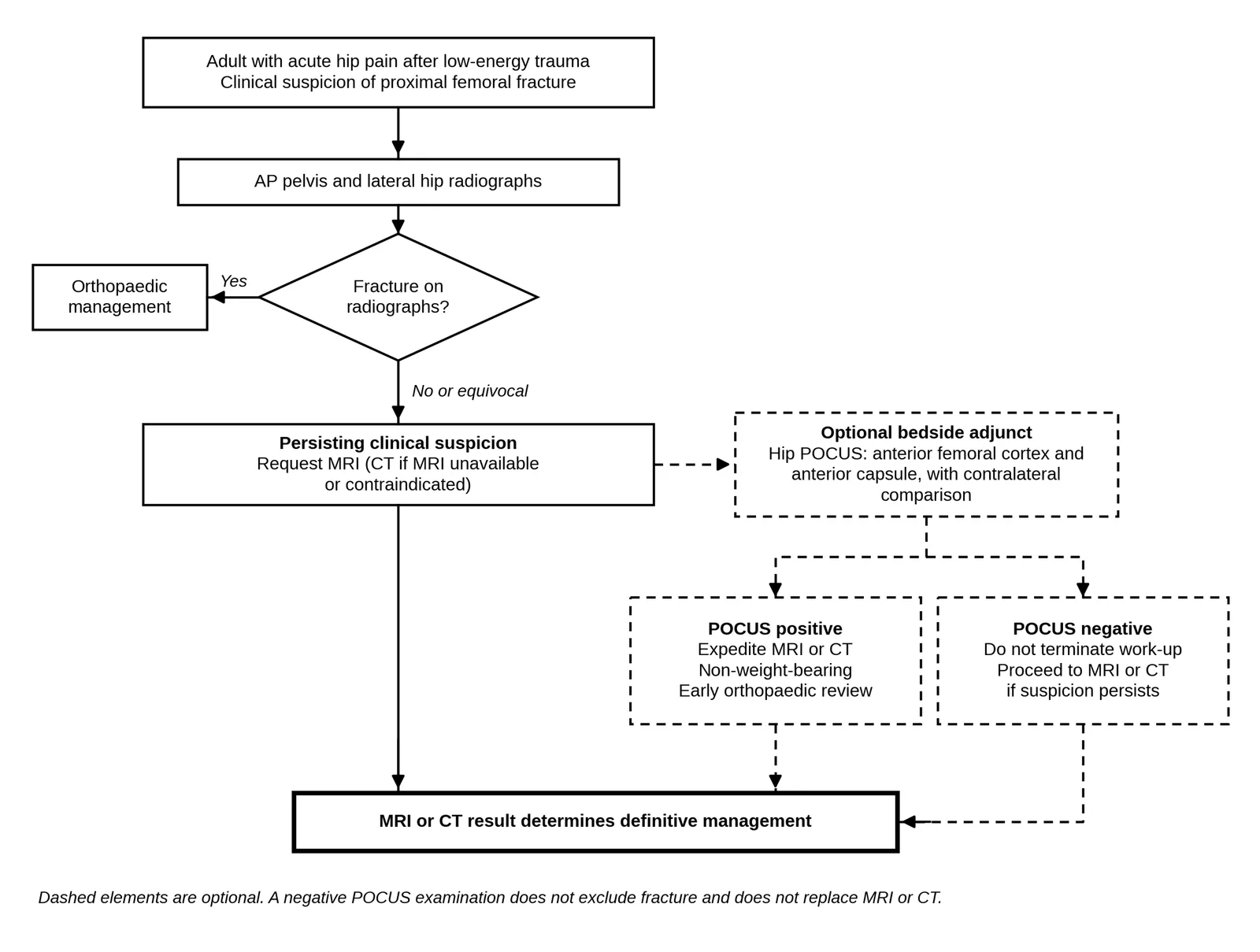
Proposed diagnostic pathway POCUS is optional and runs alongside, not before, the arrangement of advanced imaging; neither result removes the need for MRI or CT when suspicion persists. POCUS: point-of-care ultrasound; MRI: magnetic resonance imaging; CT: computed tomography; AP: anteroposterior

The examination may fit existing workflows, because clinicians performing an ultrasound-guided femoral nerve block, which reduces preoperative opioid use [26], already scan the groin; this is a practical point, not diagnostic evidence. A learning-curve study showed technical acquisition proficiency in a standardised proximal femur protocol after about 25 scans [27], but it assessed image acquisition in healthy participants, not the ability to diagnose acute fracture in symptomatic elderly patients. The diagnostic training burden therefore remains unknown.

Strengths and Limitations of the Evidence

All four studies were prospective, and two verified every participant with MRI. However, samples were small, only one study was wholly ED-based, only one used emergency POCUS operators, criteria and thresholds differed, one threshold was derived in-sample, technical parameters were largely unreported, no study reported inter-observer reliability, and none reported subtype-specific accuracy for extracapsular fractures or any patient-important outcome. The true accuracy of hip POCUS in routine practice is probably lower than observed, particularly sensitivity in the suspected-fracture cohorts and specificity overall.

Future Research

The priority is a consecutive ED cohort with confirmed non-diagnostic radiographs, a prespecified protocol (stated transducer, settings, planes, a cortical classification and a measured capsular threshold with contralateral comparison), emergency-clinician operators blinded to other data, universal MRI verification, subtype-specific reporting and inter-observer reliability. Impact studies should then measure time to imaging and surgery, length of stay, opioid use and missed fractures. Resource-limited settings, where ultrasound may be the only immediate cross-sectional modality, deserve specific study.

Limitations

The evidence base was small (four studies, 389 participants), and pooling was inappropriate. The five-year restriction was not applied, so a 2009 study using older equipment was included. Screening, extraction and QUADAS-2 assessment were led by a single reviewer with repeat assessment, which does not fully replace independent duplicate review. The protocol was not prospectively registered, which increases the risk of post hoc decisions. Restriction to English-language, peer-reviewed full texts may have introduced language and publication bias, which could not be assessed formally. Broadening eligibility beyond occult-restricted cohorts was needed for feasibility but limits applicability, and fracture-subtype analysis was not possible.

## Conclusions

Evidence on POCUS for suspected occult proximal femoral fracture comprises four small prospective studies (389 participants), all with important risk-of-bias or applicability concerns. Observed sensitivity was high (0.96-1.00) but is likely overestimated, and specificity varied widely (0.65-0.99) for reasons that cannot be separated from differences in criterion, operator, setting, fracture type and reference standard. Hip POCUS may be considered a rapid, radiation-free adjunct that modifies suspicion and helps prioritise advanced imaging, but not a rule-out test. Patients with persistent pain or inability to bear weight after negative or equivocal radiographs still require MRI or, if unavailable, CT, regardless of the ultrasound result. Future ED studies should use prespecified, measured criteria with defined probe and settings, blinded emergency-clinician operators, universal MRI verification, subtype-specific reporting, inter-observer reliability and patient-important outcomes.

## Appendices

**Table 7:** Database-specific search strategies WHO ICTRP: World Health Organization International Clinical Trials Registry Platform

Source	Platform	Records	Search strategy
MEDLINE	PubMed	455	("Hip Fractures"[Mesh] OR "Femoral Neck Fractures"[Mesh] OR "hip fracture*"[tiab] OR "femoral neck fracture*"[tiab] OR "proximal femoral fracture*"[tiab] OR "femoral proximal fracture*"[tiab] OR "trochanteric fracture*"[tiab] OR "occult fracture*"[tiab] OR "radiographically occult"[tiab]) AND ("Ultrasonography"[Mesh] OR "Point-of-Care Testing"[Mesh] OR ultraso*[tiab] OR sonograph*[tiab] OR echograph*[tiab] OR POCUS[tiab] OR "point of care ultrasound"[tiab] OR "bedside ultrasound"[tiab] OR "pocket-sized ultrasound"[tiab]) AND (diagnos*[tiab] OR accura*[tiab] OR sensitiv*[tiab] OR specific*[tiab] OR "predictive value*"[tiab] OR screen*[tiab])
Embase	Embase.com (Elsevier)	356	('hip fracture'/exp OR 'femoral neck fracture'/exp OR 'hip fracture':ti,ab OR 'femoral neck fracture':ti,ab OR 'proximal femoral fracture':ti,ab OR 'femoral proximal fracture':ti,ab OR 'trochanteric fracture':ti,ab OR 'occult fracture':ti,ab OR 'radiographically occult':ti,ab) AND ('echography'/exp OR 'point of care ultrasound'/exp OR ultraso:ti,ab OR sonograph:ti,ab OR echograph:ti,ab OR pocus:ti,ab OR 'bedside ultrasound':ti,ab OR 'pocket-sized ultrasound':ti,ab) AND (diagnos:ti,ab OR accura:ti,ab OR sensitiv:ti,ab OR specific:ti,ab OR 'predictive value':ti,ab OR screen*:ti,ab)
Scopus	Elsevier	241	TITLE-ABS-KEY(("hip fracture" OR "femoral neck fracture" OR "proximal femoral fracture" OR "femoral proximal fracture" OR "trochanteric fracture" OR "occult fracture" OR "radiographically occult") AND (ultraso* OR sonograph* OR echograph* OR pocus OR "point of care ultrasound" OR "bedside ultrasound" OR "pocket-sized ultrasound") AND (diagnos* OR accura* OR sensitiv* OR specific* OR "predictive value" OR screen))
Web of Science Core Collection	Clarivate	129	TS=(("hip fracture" OR "femoral neck fracture" OR "proximal femoral fracture" OR "femoral proximal fracture" OR "trochanteric fracture" OR "occult fracture" OR "radiographically occult") AND (ultraso* OR sonograph* OR echograph* OR POCUS OR "point of care ultrasound" OR "bedside ultrasound" OR "pocket-sized ultrasound") AND (diagnos* OR accura* OR sensitiv* OR specific* OR "predictive value" OR screen))
Cochrane CENTRAL	Cochrane Library (Wiley)	18	#1 MeSH descriptor: [Hip Fractures] explode all trees #2 MeSH descriptor: [Femoral Neck Fractures] explode all trees #3 ("hip fracture" OR "femoral neck fracture" OR "proximal femoral fracture" OR "trochanteric fracture" OR "occult fracture"):ti,ab,kw #4 MeSH descriptor: [Ultrasonography] explode all trees #5 (ultraso OR sonograph* OR echograph* OR POCUS OR "point of care ultrasound" OR "bedside ultrasound"):ti,ab,kw #6 (#1 OR #2 OR #3) AND (#4 OR #5)
ClinicalTrials.gov and WHO ICTRP	Registry websites	3	Condition: hip fracture OR femoral neck fracture OR occult hip fracture Intervention / other terms: ultrasound OR ultrasonography OR POCUS
